# Biological brain age and resilience in cognitively unimpaired 70‐year‐old individuals

**DOI:** 10.1002/alz.14435

**Published:** 2024-12-20

**Authors:** Anna Marseglia, Caroline Dartora, Jessica Samuelsson, Konstantinos Poulakis, Rosaleena Mohanty, Sara Shams, Olof Lindberg, Lina Rydén, Therese Rydberg Sterner, Johan Skoog, Anna Zettergren, Silke Kern, Ingmar Skoog, Eric Westman

**Affiliations:** ^1^ Division of Clinical Geriatrics Center for Alzheimer Research Department of Neurobiology, Care Sciences and Society Karolinska Institutet Huddinge Sweden; ^2^ Neuropsychiatric Epidemiology Unit Department of Psychiatry and Neurochemistry Institute of Neuroscience and Physiology Sahlgrenska Academy Centre for Ageing and Health (AGECAP) University of Gothenburg Mölndal Sweden; ^3^ McConnell Brain Imaging Centre (BIC), MNI Faculty of Medicine McGill University Montréal Quebec Canada; ^4^ Region Västra Götaland Sahlgrenska University Hospital Neuropsychiatry Clinic Gothenburg Sweden; ^5^ Department of Neuroimaging Centre for Neuroimaging Sciences Institute of Psychiatry Psychology and Neuroscience King's College London London UK

**Keywords:** Alzheimer's disease, brain age, fluid biomarkers, glucose, inflammation, resilience, sex differences, small vessel disease, vascular cognitive impairment

## Abstract

**INTRODUCTION:**

This study investigated the associations of brain age gap (BAG)—a biological marker of brain resilience—with life exposures, neuroimaging measures, biological processes, and cognitive function.

**METHODS:**

We derived BAG by subtracting predicted brain age from chronological age in 739 septuagenarians without dementia or neurological disorders. Robust linear regression models assessed BAG associations with life exposures, plasma inflammatory and metabolic biomarkers, magnetic resonance imaging, and cerebrospinal fluid biomarkers of neurodegeneration and vascular brain injury, and cognitive performance.

**RESULTS:**

Greater BAG (older‐looking brains) was associated with physical inactivity, diabetes, and stroke, while prediabetes was related to lower BAG, that is, younger‐looking brains. Physical activity mitigated the link between obesity and BAG. Greater BAG was associated with greater small vessel disease burden, white‐matter alterations, inflammation, high glucose, poorer vascular‐related cognitive domains. Sex‐specific associations were identified.

**DISCUSSION:**

Vascular‐related lifestyles and health shape brain appearance. Inflammation and insulin‐related processes may be keys to understanding vascular cognitive disorders.

**Highlights:**

- BAG, reflecting deviations from CA, can indicate resilience.
- Diabetes, stroke, and low physical activity link to “older” brains (greater BAG).
- Physical activity yielded to “younger” brains in septuagenarians with obesity.
- High cerebrovascular burden, inflammation, and glucose associate with “older” brains.
- Sex differences were detected in all BAG‐associated factors.

## 1 BACKGROUND

**FIGURE 1:**
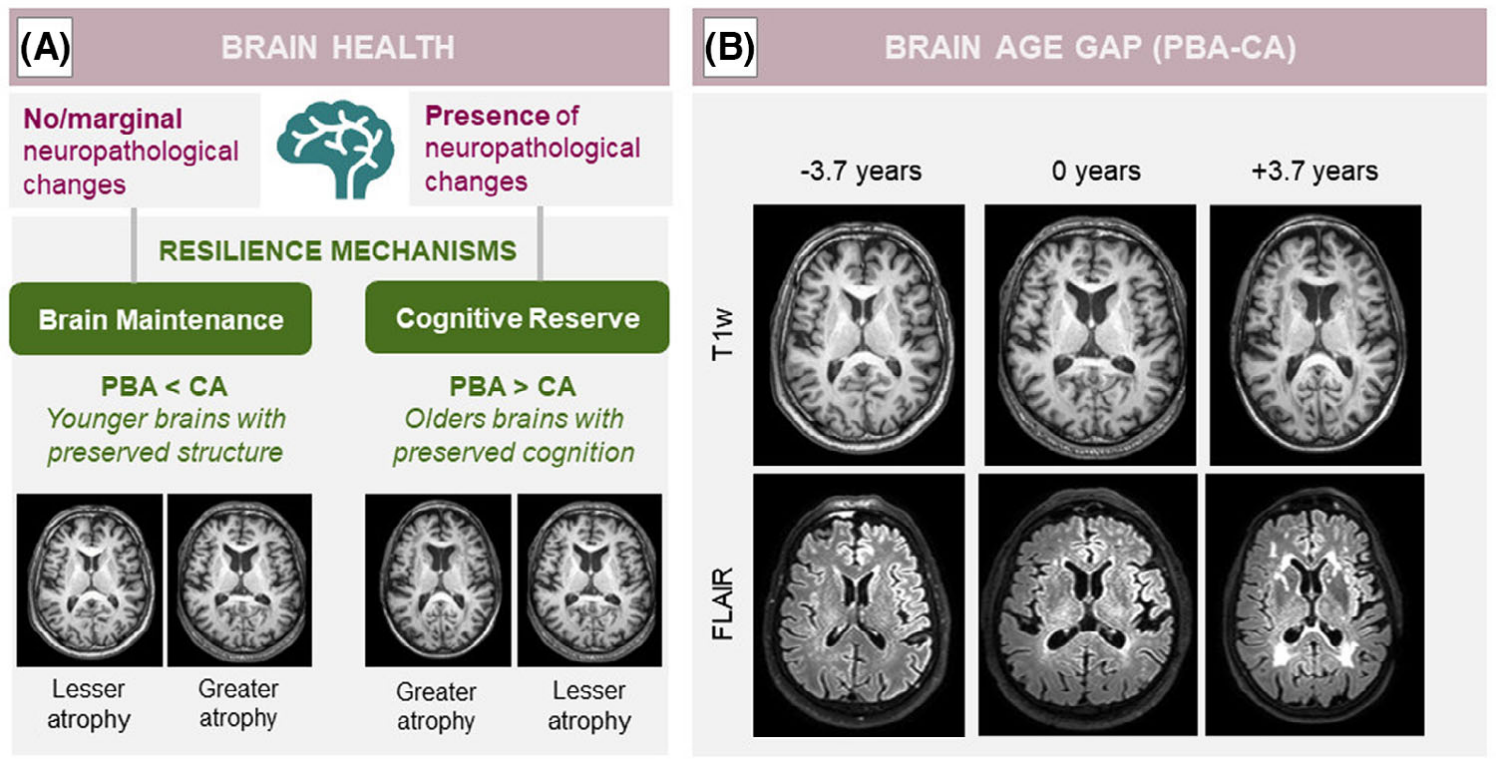
Brain age gap as potential biological markers of brain resilience. Panel A (left) presents a conceptual framework illustrating two complimentary resilience mechanisms (brain maintenance and cognitive reserve) based on the differences between an individual's PBA and CA, knows as the BAG. These differences can serve as personalized biomarkers of brain and/or cognitive health. Hypothetically, younger‐appearing brains (negative differences, PBA < CA) suggest preserved brain structure, indicative of brain maintenance. Conversely, older‐appearing brains (positive differences, PBA > CA), especially in the presence of no/minimal cognitive decline, suggest coping abilities linked to cognitive reserve. Panel B (right) shows the T1‐weighted and FLAIR brain MRIs from three participants in the Gothenburg H70 Birth Cohort Studies–Birth cohort 1944. The images illustrate cases where there are no differences between PBA and CA (BAG = 0 years), a negative difference indicative of a younger‐appearing brain (BAG = −3.7 years), and a positive difference indicative of an older‐appearing brain (BAG = +3.7 years). BAG, brain age gap; CA, chronological age; FLAIR, fluid attenuated inversion recovery; MRI, magnetic resonance imaging; PBA, predicted brain age.

Individuals show different brain aging trajectories influenced by a spectrum of negative and positive life exposures across their lifespan. Unhealthy lifestyle habits (e.g., diet, sedentary behaviors) coupled with cardiometabolic risk factors and disorders (CMDs; e.g., hypertension, heart disease, diabetes, obesity) heighten the risk of cognitive disorders, including dementia.^1^ Research consistently demonstrated the significant contribution of CMDs to brain atrophy and vascular brain injury, including markers of small vessel disease (SVD) and early changes in white matter microstructure.^2^, ^3^, ^4^ Such structural alterations can manifest as an aged brain appearance on neuroimaging. However, the processes linking structural brain alterations to clinically manifest cognitive disorders remain largely elusive. Inflammation and disruptions in glucose and lipid metabolism could be key underlying pathways. Growing literature showed that systemic inflammation is linked to CMDs,^5^ Alzheimer's disease (AD), SVD, and dementia.^6^, ^7^, ^8^ Studies examining changes in glucose and/or lipids biomarkers, alone or alongside inflammation, on the other hand, yielded mixed results.^9^, ^10^ Conversely, positive life exposures (e.g., high educational attainment, challenging jobs, social/physical/mental engagement) can influence brain aging fostering greater resilience.^11^ Resilience, encompassing the brain's ability to preserve cognitive function through structural preservation (brain maintenance) or adaptation to pathology (cognitive reserve),^12^ has traditionally posed challenges for biological measurements. Advancements in artificial intelligence facilitated the development of brain age models using the whole structural magnetic resonance imaging (MRI), capturing the resilience's core biological dimension.^13^ The brain age gap (BAG), derived from differences between an individual's predicted brain age (PBA) and chronological age (CA), can serve as a valuable biomarker of brain health.^14^ BAG is particularly promising for assessing resilience mechanisms, with negative values indicating younger brains (i.e., brain maintenance) and positive values indicating older brains (Figure 1A). In the long term, identifying the modifiable risk factors and biological processes driving BAG holds promises for uncovering neuroprotective intervention targets to improve brain health. Emerging studies have linked diastolic blood pressure, alcohol intake, and smoking to greater BAGs (older brains).^15^, ^16^ Also, white matter hyperintensity, that is, a SVD marker, has been linked to greater BAGs.^17^ However, the extent to which CMDs, vascular‐related biological processes (e.g., inflammation, glucose, and lipids alterations), neurodegenerative and vascular brain injury contribute to the BAG remains poorly understood. Also, to fully realize BAG's potential as a resilience biomarker, its role in cognitive function must be clarified. While studies consistently show that greater BAG is associated with poor cognition findings across key cognitive processes in aging are less consistent.^18^, ^19^, ^20^, ^21^

**RESEARCH IN CONTEXT**

1. **Systematic review**: We reviewed PubMed literature on resilience measures and their associations with dementia risk/protective factors, magnetic resonance imaging (MRI) markers of neurodegenerative and cerebrovascular disease, plasma biomarkers, and cognitive function. Most studies used proxy‐based/residual approaches to measure resilience mechanisms (e.g., cognitive reserve and brain maintenance), overlooking key biological aspects, which can be captured by the brain age gap (BAG), a potential biomarker of resilience. The role of life exposures, inflammation, insulin‐related and lipid metabolism (biological processes), and vascular brain injury on BAG remain poorly investigated.
2. **Interpretation**: Cardiometabolic disorders, inflammation, small vessel disease (SVD; white‐matter hyperintensity, lacunes), and insulin‐related dysregulation influence brain age deviations from chronological age (CA). Favorable lifestyles, like physical activity, may help preserve brain integrity, thus “younger‐looking” brains.
3. **Future directions**: Longitudinal studies should clarify how cardiometabolic disorders, inflammation, glucose metabolism, and vascular brain injury interact to influence BAG and cognitive outcome, while accounting for sex differences.

This study investigated associations between a potential biological marker of brain resilience (BAG) and life exposures (i.e., lifestyle factors, CMDs), biological processes (i.e., inflammation, glucose, and lipids alterations), brain injury related to neurodegenerative and vascular disease (i.e., overall, AD, and cerebrovascular neuroimaging markers) that are hypothesized to influence brain aging (Figure S1). We also examined the associations between BAG and domain‐specific cognitive functioning. Finally, we compared how these associations varied between sexes, defined biologically at birth. We applied an in‐house developed deep‐learning brain age model to minimally processed structural imaging data to derive BAG in cognitively unimpaired septuagenarians.

## 2 METHODS

### 2.1 Study design and participants

The study sample, drawn from the Swedish population‐based Gothenburg H70 Birth Cohort Studies, examining 70‐year‐olds, born in 1944, conducted during 2014–2016. The examined cohort comprised 1203 individuals (72% response rate), living in Gothenburg, and identified through the Swedish Tax Agency's population register. Detailed study design and procedures are in Rydberg Sterner and coll.^22^ All 1203 participants were invited to undergo brain MRI; 381 declined, and 31 were not eligible due to contraindications. Consequently, 791 participants (65.7% response rate) underwent brain MRI. Of these, we excluded individuals with unsuccessful FreeSurfer processing (*n* = 3), those who did not pass FreeSurfer image segmentation quality control (*n* = 29), those with dementia (*n* = 9) and other neurological disorders (*n* = 11) (Figure S2). Thus, the current study included 739 participants without dementia or neurological disorders. Cerebrospinal fluid (CSF) data were available for a subset of 283 individuals. Ethical approval for the H70 study was granted by the Regional Ethical Review Board in Gothenburg.

### 2.2 Neuroimaging acquisition protocol and processing

Details on the scanning protocol and image processing have been previously described.^20^ In brief, participants were scanned on a 3.0T scanner (Philips Medical Systems). The protocol included a 3D T1‐weigthed turbo field echo sequence (repetition time [RT] = 7.2 ms, echo time [TE] = 3.2 ms, flip angle = 9°, matrix size = 250 mm × 250 mm, field of view = 256 × 256, slice thickness = 1.0 mm); a 3D fluid‐attenuated inversion recovery [FLAIR] sequence (RT = 48,000 ms, TE = 280 ms, TI = 1650 ms, flip angle = 90°, number of slices = 140, matrix size = 250 mm × 237 mm, slice thickness = 2.0 mm); a susceptibility‐weighted imaging (SWI) sequence (RT = 14.59–17.60 ms., TE = 20.59–24.99 ms, flip angle = 10°, matrix size = 229 mm × 222 mm, slice thickness = 1.0 mm); and a diffusion tensor imaging (DTI) sequence encoded with 1 *b*‐value shell: 800ks/mm^2^, along with 32 directions and 1 *b* = 0 image (RT = 7340 ms, ET = 83 ms, flip angle = 90°, matrix size = 112 mm × 112 mm, field of view = 224 × 224, slice thickness = 3.0 mm). Neuroimaging data were automatically pre‐processed using FreeSurfer version 7.2 (https://surfer.nmr.mgh.harvard.edu/), SPM (https://www.fil.ion.ucl.ac.uk/spm/), and FSL's Diffusion Toolbox (https://fsl.fmrib.ox.ac.uk/fsl/fslwiki). All MRI data were quality controlled by a neuroimaging expert (O.L.) following a previously described procedure.^23^ MRI data were managed and processed through theHiveDB system^24^ at Karolinska Institutet, Stockholm.

### 2.3 Brain age prediction and BAG

Biological brain age is a theoretical concept of a measurement developed to track the state of a person's biophysiological brain aging, to unveil why individuals with the same CA present different atrophy patterns (aging heterogeneity).

The model in the current study is based on a Convolutional Neural Network, trained on 18,843 raw T1‐weighted brain MRIs of cognitively unimpaired individuals, with an average age of 65.4 ± 9.0 years, from 7 different cohorts (AddNeuroMed, ADNI, AIBL, GENIC, UK Biobank and all waves/branches of the Gothenburg birth cohorts—born 1930 and 1944), using a 10‐fold cross‐validation approach. The whole brain images were only rigidly registered to the Montreal Neuroscience Institute (MNI) atlas space before being used as input to the model. More details about the model can be found in Dartora et al.^25^ The BAG was calculated by subtracting the participant's CA from the PBA. Higher BAG scores indicated older brains. Lower BAG scores indicated younger brains, thus preserved brain structure (Figure 1).

### 2.4 Data collection

Participants took part in a comprehensive general assessment at the Neuropsychiatry outpatient clinic at Sahlgrenska University Hospital in Gothenburg, Sweden, or in their homes. Information on sociodemographic and lifestyle factors, medical conditions, and cognitive function were collected by research nurses and physicians through semi‐structured interviews and clinical examinations. Blood samples were taken from all participants and analyzed following the standard lab routines at Mölndal Clinical Neurochemistry Laboratory, Sahlgrenska University Hospital. A subset of participants consented to collecting CSF samples via lumbar punctures. Detailed assessments procedure has been described elsewhere.^22^ Below, we summarize the operationalization of potential determinant factors contributing to BAG in the current study.

### 2.5 Sociodemographic, lifestyle, and medical conditions

2.5

This included sex (men vs. women), education (primary/lower secondary education with <9 years of schooling vs. secondary school or higher education), smoking (never vs. current/former), at‐risk alcohol consumption (>98 g/week), body mass index (BMI; underweight [<20 kg/m2], normal [20 to <25 kg/m^2^], overweight [25 to < 30 kg/m^2^], obese [≥30 kg/m^2^]), and physical activity (inactive/light vs. active including regular activity or hard physical training).^26^ Medical conditions included hypertension, heart disease, prediabetes and diabetes, stroke/transient ischemic attack (TIA), and depression. Diagnoses of medical conditions (hypertension, heart disease, prediabetes and diabetes, stroke/TIA, and depression) were based on self‐reported medical history, medical records from the Swedish National Patient Register, medication use, and biochemical measures (for details on the assessments and diagnostic criteria, see Table S1). DNA was extracted from blood samples and apolipoprotein E (*APOE*) was genotyped (any ε4 allele carriers vs. non‐carriers).

### 2.6 Structural neuroimaging markers of neurodegenerative and vascular brain injury

2.6

The mean cortical thickness, serving as indicator of overall neurodegeneration, was derived from T1‐weighted images through FreeSurfer 7.2. To capture AD neurodegeneration, a specific signature was computed by averaging the bilateral cortical thickness of the entorhinal, inferior temporal, middle temporal, and fusiform regions from the Desikan‐Killiany atlas.^27^ A brain resilience signature was also calculated by averaging the bilateral thickness of the rostral anterior cingulate, caudal anterior cingulate, superior frontal, and temporal pole regions.^27^ AD and resilience signatures were adjusted for the region of interest's (ROI) surface areas to account for differences in size.

Cerebral SVD markers were visually evaluated by an expert radiologist (S.S.) using standard rating scales and according to the *Standards for Reporting Vascular Changes on Neuroimaging* (STRIVE) guidelines.^28^ SVD markers comprised white matter hyperintensities (WMHs) assessed with the Fazekas scale applied on FLAIR dichotomized into none/punctuate (Fazekas 0–1) versus confluent (Fazekas 2–3); lacunes (3–15 mm hypointensities in T1‐weighted and FLAIR, or hyperintensities in T2‐weighted) dichotomized into none versus present (≥1); cerebral microbleeds (CMBs; Microbleed Anatomical Rating Scales applied on SWI) categorized into none versus present (≥1); perivascular spaces (PVS) in the centrum semiovale and basal ganglia (Mac Lullich's Rating Scale applied on T2‐weighted) dichotomized into 0–10 versus ≥11–40. Additionally, large infarctions (> 15 mm) were assessed through visual inspection. A summary SVD score (ranging 0–4), reflecting cerebrovascular burden, was generated by awarding one point if the following SVD markers were present: confluent WMHs (Fazekas's score 2–3), ≥ 1 lacunes, ≥ 1 CMBs, and ≥ 11–40 PVS in basal ganglia.^29^ To account for low power, SVD scores 3 (*n* = 26) and 4 (*n* = 2) were combined for the analysis.

In addition to visual assessment, WMH volume was quantified on FLAIR and automatically segmented through the Lesion Segmentation Toolbox (LST 2.0.15) in the SPM software. Adjustment for SPM‐derived total intracranial volume was performed using a residuals approach.^30^ The mean fractional anisotropy (FA) measure was obtained from DTI scans, which were analyzed using the FMRIB's Diffusion Toolbox from FSL. This measurement serves as an indicator of white matter integrity based on the directionality of water diffusion along the white matter tracts. Lower FA values indicate white‐matter microstructural abnormalities.

### 2.7 Plasma inflammation, glucose, and lipids biomarkers

Peripheral blood samples were obtained for laboratory tests, with cutoffs for altered biomarker status aligned with the Mölndal Clinical Neurochemistry Laboratory standards. Biomarkers indicating systemic and vascular‐related inflammation included C‐reactive protein (CRP) levels ≥5 mg/L and homocysteine levels >13.5 µmol/L. Because of the substantial variability in CRP levels within the altered group, this was further stratified into low (CRP ≥5–7 mg/L) and high (CRP ≥8 mg/L) subgroups based on the median CRP levels among individuals with altered CRP status. Glucose and lipid biomarkers encompassed high glucose levels ≥7.0/11.1 (fasting/non‐fasting) mmol/L, high total cholesterol levels ≥7.8 mmol/L, high triglycerides levels ≥2.6 mmol/L, reduced high‐density lipoprotein cholesterol (HDL‐c) levels ≤1.0 mmol/L, and high low‐density lipoprotein cholesterol (LDL‐c) levels ≥5.3 mmol/L.

### 2.8 CSF biomarkers

Adhering to the standard clinical procedure, lumbar punctures were carried out in the morning to collect CSF samples from a subset of 283 cognitively unimpaired participants. Detailed information on the assay characteristics and methods for amyloid‐β42 (Aβ42), phosphorylated tau at threonine 181 (p‐tau), total‐tau (t‐tau), neurofilament light (NFL), neurogranin (Ng), and CSF/serum albumin ratio (CSF/serum Alb) as part of routine clinical diagnostics has been previously outlined.^22^ Pathological CSF biomarkers of AD‐specific and non‐specific neurodegeneration were defined as Aβ42 ≤ 530 pg/mL, p‐tau ≥80 pg/mL, and t‐tau ≥350 pg/mL.^31^ Elevated CSF levels of NFL and Ng typically indicate increased neurodegeneration. A CSF/serum Alb ≥ 10.2 indicated blood‐brain barrier (BBB) dysfunction. All cutoffs were in accordance with the clinical reference standards at Sahlgrenska University Hospital.

### 2.9 Assessment of cognitive function

Clinical diagnoses of dementia were made according to the Diagnostic and Statistical Manual of Mental Disorders–3rd Revised edition using information from the neuropsychiatric examination and the key informant interview at a consensus meeting by psychiatrists. Dementia was used as an exclusion criteria in our study. All participants were administered a cognitive test battery by trained staff following standard procedures to assess five cognitive domains: episodic memory (Memory in Reality [free recall and 12‐object delayed recall] and Thurstone's picture memory), attention/speed (Figure Identification and Digit Span Forward), executive function (Digit Span Backward and Figure Logic), verbal fluency (phonemic and semantic), and visuospatial abilities (Koh's block test).^32^ Tests’ raw scores were *z*‐standardized and averaged within each domain. A global cognitive performance composite score (G‐score) was then derived by averaging *z*‐scores across all five domains.

### 2.10 Statistical analysis

In the descriptive analysis, we compared participants’ characteristics (sociodemographic, lifestyle, health‐ and brain‐related, cognitive, and biomarkers) by sex using a *χ* ^2^ test for categorical variables, and either a *t*‐test or quantile regression for normally or non‐normally distributed continuous variables, respectively.

Robust linear regression models (RLRMs; Stata's rreg command) were employed to quantify associations of life exposures, neuroimaging measures of neurodegeneration and vascular brain pathology, and plasma biomarkers with BAG (outcome), as well as between BAG (exposure) and cognitive outcomes. RLRMs address outliers’ impact, providing β‐coefficients and 95% confidence intervals (CIs) less influenced by extreme data points without discarding valuable information. The RLRMs were designed to ensure the number of participants far exceeded the number of independent variables, providing sufficient power to estimate medium‐sized associations.

Three distinct sub‐models were executed. First, a *life exposure model* included sociodemographic (sex and education), lifestyle (smoking, alcohol risk consumption, BMI, physical inactivity), medical conditions (hypertension, diabetes status, stroke/TIA, depression), and AD genetic predisposition (*APOE*‐ɛ4 allele). Second, a *neurodegenerative* and a *cerebrovascular model* examined brain tissue injury's association with BAG, including mean cortical thickness, AD signature, resilience signature, SVD score, and DTI‐based FA. Third, a *plasma biomarkers model* explored the potential influence of systemic inflammation, altered glucose and lipids metabolism on BAG, assessing CRP, homocysteine, glucose, total cholesterol, triglycerides, HDL‐c, and LDL‐c. Each variable was initially entered separately (model 1) and then simultaneously (model 2). Model 1 was nested within model 2 to avoid issues related to multiple comparisons. For the neurodegeneration, mean cortical thickness, AD signature, and resilience signature were not included in the same model due to multicollinearity. Thus, *p*‐values were Bonferroni‐adjusted (*α*/*n* [0.05/4]) ≥0.013).[Table alz14435-tbl-0001]

We also assessed associations between BAG and cognitive function using six education‐adjusted RLRMs. Composite cognitive scores, including a global cognition (G‐score) and five cognitive domains, were analyzed as separate outcomes, with BAG as independent variable. Bonferroni correction was applied for multiple comparisons, setting statistical significance at *p* ≥ 0.008 (*α*/*n* [0.05/6]).

Furthermore, sex‐stratified analyses were conducted to identify sex‐related differences in BAG determinants for males and females.

In supplementary analysis, associations between CSF biomarkers (AD‐specific, neurodegenerative, and BBB alterations) and BAG were examined in the subset with CSF sampling (*n* = 283). Finally, we also analyzed associations between individual markers of SVD, used to generate the SVD score, and BAG.

Multicollinearity was assessed using the variance inflation factor (VIF) for all predictors in the fully adjusted models. VIF consistently below 2.9, affirming the feasibility of including these variables simultaneously. Statistical significance was defined as a *p*  <  0.05 for two‐sided tests, and Bonferroni‐adjusted threshold were applied when necessary for multiple comparisons. All analyses were conducted using Stata‐SE 18.

## 3 RESULTS

### 3.1 Characteristics of the study population

Table 1 presents sociodemographic features, lifestyle, vascular risk factors, medical conditions, neuroimaging markers, plasma and CSF biomarkers, and cognitive function in the overall sample and stratified by sex. Among the 739 septuagenarians, 47.3% were male and 52.6% were female. Median (interequartile range [IQR]) was 70.7 (70.6 to 71) years for CA, 71.1 (70.3 to 72.1) years for PBA, and 0.3 (−0.5 to 1.4) years for BAG, comparable between sexes. Approximatively 90% had secondary or higher education. Median Mini Mental State Examination (MMSE) score was 29, indicating high cognitive function. Cardiometabolic conditions ranged from 18% for heart disease to 70% for hypertension. About 38% had at least one SVD, and 4%–17% had altered CRP/homocysteine, glucose, or lipids levels. Within the CSF sample (*n* = 283, 145 [51.2%] males, 138 [48.8%] females), altered Aβ42, p‐tau, and t‐tau were detected in 47%, 6%, and 32%, respectively. About 9% had altered CSF/serum albumin ratio.

**TABLE 1 alz14435-tbl-0001:** Characteristics of study participants from the Gothenburg H70‐Birth Cohort 1944.

		By biological sex at birth		
Characteristics	Total (*n* = 739)	Male (*n* = 350)	Female (*n* = 389)	*p*‐value^a^
Chronological age	70.7 (70.6–71)	70.7 (70.6–71)	70.7 (70.6–71)	1.000
Predicted brain age	71.1 (70.3–72.1)	71.1 (70.3–72.1)	71.2 (70.3–72.2)	0.438
Brain age gap (PBA‐CA)	0.3 (−0.5–1.4)	0.2 (−0.5–1.3)	0.4 (−0.5–1.5)	0.121
Education (years)	13 (10–16)	13 (10–17)	13 (10–16)	1.000
Primary school	82 (11.1)	50 (14.3)	32 (8.2)	0.003
Secondary school	366 (49.5)	153 (43.7)	213 (54.8)	
Higher education	291 (39.4)	147 (42.0)	144 (37.0)	
*APOE* ɛ4 status				
Non‐carriers	482 (66.7)	226 (65.1)	256 (68.1)	0.400
Carriers	241 (33.3)	121 (34.9)	120 (31.2)	
MMSE score	29 (29–30)	29 (28–30)	30 (29–30)	<0.001
**Lifestyle factors**				
Current/former smoking	448 (60.8)	214 (61.3)	234 (60.3)	0.779
Alcohol risk consumption	230 (31.2)	143 (40.9)	87 (22.4)	<0.001
BMI (kg/m^2^)	25.3 (23.0–28.0)	25.7 (23.6–28.1)	24.9 (22.4–28.0)	0.012
Underweight (<20)	38 (5.2)	12 (3.4)	26 (6.7)	0.002
Normal (≥20–25)	306 (41.5)	132 (37.7)	174 (44.9)	
Overweight (≥25–30)	285 (38.6)	159 (45.4)	126 (32.5)	
Obese (≥30)	109 (14.8)	47 (13.4)	62 (16.0)	
Physical inactivity	25 (3.5)	14 (4.1)	11 (2.9)	0.399
**Medical conditions**				
Hypertension	513 (69.4)	243 (69.4)	270 (69.4)	0.995
Heart disease	130 (17.6)	79 (22.6)	51 (13.1)	<0.001
Diabetes status				
Normoglycemia	315 (42.6)	118 (33.7)	197 (50.6)	<0.001
Prediabetes	318 (43.0)	163 (46.6)	155 (39.9)	
Diabetes	106 (14.3)	69 (19.7)	37 (9.5)	
Stroke/TIA	55 (7.4)	21 (6.0)	34 (8.7)	0.156
Depression	59 (8.0)	23 (6.6)	36 (9.3)	0.176
**Neuroimaging markers**				
Mean cortical thickness (mm)	2.36 (2.30–2.4)	2.35 (2.30–2.40)	2.36 (2.31–2.41)	0.047
AD signature (mm)^b^	2.73 (2.67–2.79)	2.73 (2.67–2.79)	2.72 (2.67–2.78)	0.091
Resilience signature (mm)^b^	2.58 (2.50–2.64)	2.56 (2.48–2.62)	2.59 (2.52–2.65)	<0.001
Small vessel disease score				
0	459 (62.1)	210 (60.0)	249 (64.0)	0.018
1	185 (25.0)	82 (23.4)	103 (26.5)	
2	67 (9.1)	38 (10.9)	29 (7.46)	
≥3	28 (3.8)	20 (5.7)	8 (2.1)	
WMHV (mm^3^)	4.67 (2.24–8.04)	3.54 (1.0–7.6)	5.18 (3.52–8.4)	<0.001
WMHs burden (Fazekas score)				
None/punctate (0–1)	622 (84.6)	287 (82.2)	335 (86.8)	0.087
Confluent (2–3)	113 (15.4)	62 (17.8)	51 (13.2)	
Lacunes	53 (7.2)	33 (9.4)	20 (5.2)	0.025
Perivascular spaces (≥11–40)				
Centrum semiovale	539 (73.2)	264 (75.6)	275 (71.1)	0.161
Basal ganglia	160 (21.7)	89 (25.5)	71 (18.4)	0.019
Large infarction (>15 mm)	9 (1.2)	3 (0.9)	6 (1.6)	0.397
Cerebral microbleeds	79 (10.8)	35 (10.0)	44 (11.4)	0.549
Fractional anisotropy (DTI)	0.35 (0.34–0.37)	0.35 (0.33–0.36)	0.35 (0.34–0.37)	<0.001
**Inflammation, glucose and lipid biomarkers** ^c^				
C‐reactive protein	1.0 (0.5–3.0)	1.0 (0.5–2.0)	1.0 (0.5–3.0)	1.000
Altered (≥5 mg/L)	98 (13.6)	40 (11.6)	58 (15.3)	0.141
Low (≥5–7 mg/L)	43 (6.0)	19 (5.5)	24 (6.4)	0.285
High (≥8 mg/L)	55 (7.6)	21 (6.1)	34 (9.0)	
Homocysteine	12.4 (10.3–14.7)	13.2 (11.1–15.3)	11.7 (9.7–14.3)	<0.001
Normal (<16 µmol/L)	599 (82.6)	276 (79.8)	323 (85.2)	0.053
High (≥16 µmol/L)	126 (17.4)	70 (20.2)	56 (14.8)	
Glucose (mmol/L)	5.7 (5.3–6.2)	5.8 (5.4–6.5)	5.6 (5.2–6.0)	0.001
High (≥7.0/11.1 mmol/L fasting/non‐fasting)	90 (12.3)	60 (17.2)	30 (7.8)	<0.001
Total cholesterol	5.5 (4.7–6.3)	5.1 (4.4–5.8)	5.9 (5.1–6.7)	<0.001
Normal (<7.8 mmol/L)	707 (96.1)	342 (98.0)	365 (94.3)	0.010
High (≥7.8 mmol/L)	29 (3.9)	7 (2.0)	22 (5.7)	
Triglycerides	1.1 (0.8–1.5)	1.1 (0.8–1.5)	1.1 (0.8–1.4)	1.000
Normal (<2.6 mmol/L)	705 (95.8)	330 (94.6)	375 (96.9)	0.114
High (≥2.6 mmol/L)	31 (4.2)	19 (5.4)	12 (3.0)	
High‐density cholesterol	1.6 (1.3–2.0)	1.4 (1.2–1.7)	1.9 (1.6–2.3)	<0.001
Normal (>1.0 mmol/L)	678 (92.1)	303 (86.2)	375 (96.9)	<0.001
Reduced (≤1.0 mmol/L)	58 (7.9)	46 (13.2)	12 (3.1)	
Low‐density cholesterol	3.5 (2.8–4.2)	3.3 (2.6–4.0)	3.6 (2.9–4.4)	0.004
Normal (<5.3 mmol/L)	698 (94.8)	337 (96.6)	361 (93.3)	0.045
High (≥5.3 mmol/L)	38 (5.2)	12 (3.4)	26 (6.7)	
**CSF biomarkers (** *n* ** = 283)** ^c^				
β‐amyloid 42 (pg/mL)	543 (408–665)	532 (399–647)	573 (427–677)	0.134
Altered (≤530 pg/mL)	131 (46.8)	71 (49.7)	60 (43.8)	0.326
P‐tau (ng/L)	47 (38–56)	48 (38–56)	47 (38–55)	0.601
Altered (≥80 pg/mL)	16 (5.7)	8 (5.5)	8 (5.8)	0.918
Total tau (ng/L)	299 (248–384)	309 (245–391)	292 (249–360)	0.289
Altered (≥350 pg/mL)	90 (31.8)	54 (37.2)	36 (26.1)	0.044
Neurofilament light (pg/mL)	728 (558–982)	743 (581–976)	708 (537–884)	0.433
Neurogranin (pg/mL)	196 (157–233)	194 (155–232)	198 (161–236)	0.714
CSF/serum albumin ratio	6.1 (4.9–7.9)	6.7 (5.3‐8.4)	5.7 (4.5‐6.8)	0.002
Altered (≥10.2)	26 (9.2)	21 (14.5)	5 (3.6)	0.002
**Cognitive function**				
Global cognition	0.04 ± 0.61	−0.02 ± 0.66	0.10 ± 0.57	0.011
Episodic memory	0.04 ± 0.76	−0.18 ± 0.80	0.23 ± 0.65	<0.001
Attention/speed	0.05 ± 0.77	0.02 ± 0.76	0.07 ± 0.78	0.410
Executive function	0.04 ± 0.79	0.05 ± 0.83	0.03 ± 0.77	0.804
Verbal fluency	0.003 ± 0.85	−0.16 ± 0.87	0.15 ± 0.80	<0.001
Visuospatial abilities	0.09 ± 0.10	0.19 ± 1.02	−0.01 ± 0.96	0.007

*Note*: Data presented as number (proportion, %), means ± standard deviations (*p*‐value calculated with *t*‐test), or median (25th–75th percentile; *p*‐value calculated with quantile regression) for non‐normally distributed variables. Missing data: 16 for *APOE*, 3 for MMSE, 2 for smoking status, 1 for alcohol risk consumption, 1 for BMI, 14 for physical inactivity, 1 for depression, 1 for WMHV, 4 for WMHs burden, 1 for lacunes, 3 for perivascular spaces, 4 for cerebral microbleeds, 2 for large infarcts, 35 for fractional anisotropy, 14 for homocysteine, 5 for glucose, 3 for total cholesterol, 3 for triglycerides, 3 for high‐density cholesterol, 3 for low density cholesterol, and 3 for β‐amyloid 42 (in the CSF subsample).

Abbreviations: AD, Alzheimer's disease; *APOE*, apolipoprotein gene; BMI, body mass index; CA, chronological age; CSF, cerebrospinal fluid; DTI, diffusion tensor imaging; MMSE, Mini Mental State Examination; MRI, magnetic resonance imaging; PBA, predicted brain age; TIA, transient ischemic attack; WMHs, white matter hyperintensities; WMHV, white matter hyperintensities volume.

^a^ *p*‐Value < 0.05 were considered statistically significant.

^b^ AD (mean thickness of entorhinal cortex, inferior temporal, middle temporal, and fusiform gyrus) and resilience (mean thickness of rostral anterior cingulate, caudal anterior cingulate, superior frontal, and temporal pole) signatures are adjusted for the cortical surface areas.

^c^ Cutoffs were used according to the Sahlgrenska University Hospital laboratory values (CRP, homocysteine, total cholesterol, triglycerides, high‐density cholesterol, and CSF biomarkers) or the American Diabetes Association (glucose). In participants with abnormal CRP, the continuous CRP levels were dichotomized according to the median CRP level value.

Females had lower primary schooling and higher secondary education. Males exhibited less favorable lifestyle, with higher alcohol risk consumption, overweight, and physically inactivity. They were more likely to have heart disease or diabetes, elevated homocysteine and glucose levels, and reduced HDL‐c. Females, on the other hand, showed higher total cholesterol and LDL‐c. In the brain, males had lower cortical thickness, higher prevalence of SVD—particularly lacunes and PVS in the basal ganglia—but lower WMH volume. Cognitively, males scored lower in episodic memory and verbal fluency, whereas female performed lower in visuospatial tasks. In the CSF subsample, males were more likely to have altered t‐tau and CSF/serum albumin ratio.

### 3.2 Associations between BAG and life exposure

Table 2 displays the mean differences in BAG (RLRMs’ β‐coefficients) with respect to sociodemographic and risk factors. Unadjusted RLRMs, revealed that greater BAG, indicating older brains, was associated with physical inactivity, diabetes, and stroke/TIA. Conversely, prediabetes was associated with lower BAG, suggesting younger brains. After adjusting RLRM for all risk factors simultaneously, the observed associations remained consistent, except for prediabetes and obesity, which shifted to being associated with lower BAG (β = −0.45 [95% CI −0.80, −0.07], *p* = 0.019). We hypothesized that this paradoxical association was influenced by healthier lifestyle, particularly physical activity.

**TABLE 2:** Associations of brain age gap with life exposures.

	Model 1^a^		Model 2^b^	
Life exposures	β (95% CI)	*p*‐value	β (95% CI)	*p*‐value
Biological sex				
Male	Reference		Reference	
Female	0.12 (−0.10, 0.33)	0.296	0.27 (−0.03, 0.56)	0.076
Education, years				
Primary school	Reference		Reference	
Secondary school	−0.27 (−0.63, 0.09)	0.142	−0.41 (−0.87, 0.04)	0.078
Higher education	−0.17 (−0.54, 0.19)	0.353	−0.37 (−0.84, 0.10)	0.122
Smoking				
Never	Reference		Reference	
Former/current	0.01 (−0.21, 0.23)	0.906	−0.001 (−0.28, 0.28)	0.997
Alcohol risk consumption				
No	Reference		Reference	
Yes	0.12 (−0.11, 0.35)	0.309	0.25 (−0.05, 0.55)	0.107
Body mass index				
Normal (≥20–25)	Reference		Reference	
Underweight (< 20)	0.38 (−0.12, 0.89)	0.137	0.47 (−0.23, 1.05)	0.210
Overweight (≥25–30)	−0.19 (−0.43, 0.05)	0.125	−0.03 (−0.34, 0.29)	0.867
Obese (≥30)	−0.21 (−0.53, 0.12)	0.209	−0.61 (−0.80, −0.07)	0.009
Physical activity				
Normal	Reference		Reference	
Inactive	1.59 (1.00, 2.18)	<0.000	1.75 (1.13, 2.38)	<0.000
Hypertension				
No	Reference		Reference	
Yes	0.03 (−0.27, 0.32)	0.857	0.05 (−0.26, 0.37)	0.758
Heart disease				
No	Reference		Reference	
Yes	0.09 (−0.19, 0.38)	0.522	0.14 (−0.24, 0.51)	0.474
Diabetes status				
Normoglycemia	Reference		Reference	
Prediabetes	−0.27 (−0.51, −0.04)	0.021	−0.06 (−0.37, 0.25)	0.691
Diabetes	0.35 (0.03, 0.68)	0.034	0.78 (0.32, 1.24)	0.001
Stroke/TIA				
No	Reference		Reference	
Yes	0.41 (−0.01, 0.82)	0.053	0.53 (0.10, 0.96)	0.046
Depression				
No	Reference		Reference	
Yes	0.09 (−0.30, 0.49)	0.647	−0.01 (−0.51, 0.49)	0.982
*APOE* ɛ4 status				
Non‐carriers	Reference		Reference	
Carriers	0.12 (−0.11, 0.36)	0.296	0.11 (−0.18, 0.40)	0.455

Abbreviations: APOE, apolipoprotein E; CI, confidence interval; TIA, transient ischemic attack.

^a^ Model 1: Robust linear regression models included each risk factor separately.

^b^ Model 2: Robust linear regression model included all factors simultaneously. The average variance inflation factor was 1.4 (range 1.0–2.9), indicating absence/very low collinearity.

Therefore, we explored this hypothesis by incorporating a cross‐term product between obesity and physical activity in the multi‐adjusted RLRM for all risk factors. No multiplicative interaction (*p* = 0.546) was detected. Next, we investigated whether physical activity modified the obesity‐BAG relationship by incorporating a categorical variable combining obesity and physical activity (Table S2). Participants with obesity and physically active exhibited the lowest BAG, not only when compared to the group with obesity and physical inactivity (β = −1.93 [95% CI −2.86, −0.99], *p* < 0.001), but also when compared to participants with normal BMI yet physically inactive (β = −1.92 [95% CI −3.15, −0.69], *p* = 0.002).

### 3.3 Associations between BAG and structural neuroimaging markers

Tables 3 and 4 display the mean difference in BAG concerning neuroimaging markers of neurodegeneration and vascular brain injury. Greater thicknesses in the whole brain (β = −3.16 [95% CI −4.54, −1,78], *p* < 0.001), AD (β = −1.86 [95% CI −3.02, −0.71], *p* = 0.002), and resilience (β = −1.92 [95% CI −2.96, −0.89], *p* < 0.001) signature regions were associated with lower BAG (Table 3). A SVD score ≥2 (i.e., high SVD burden) and lower values of DTI‐derived FA (i.e., lower white‐matter microstructural integrity) were both associated with greater BAG (Table 4). When adjusted for each other in the same model, results for FA remained consistent, whereas the SVD score was no longer statistically significant. Further adjusting for sex did not alter results (Tables 3 and 4, models 2).

**TABLE 3:** Associations of brain age gap with structural brain MRI markers of neurodegeneration and resilience.

	Model 1^a^		Model 2^b^	
Brain MRI markers (mm)	β (95% CI)	*p*‐value^d^	β (95% CI)	*p*‐value^d^
Mean cortical thickness	−3.16 (−4.54, −1.78)	<0.001	−3.25 (−4.64, −1.86)	<0.001
AD signature^c^	−1.86 (−3.02, −0.71)	0.002	−1.80 (−2.96, −0.65)	0.002
Resilience signature^c^	−1.92 (−2.96, −0.89)	<0.001	−2.06 (−3.11. −1.01)	<0.001

Abbreviations: AD, Alzheimer's disease; CI, confidence interval; MRI, magnetic resonance imaging.

^a^ Model 1: Robust linear regression models included the three MRI markers separately.

^b^ Model 2: Robust linear regression model included model 1 + biological sex.

^c^ AD (mean thickness of entorhinal cortex, inferior temporal, middle temporal, and fusiform gyrus) and resilience (mean thickness of rostral anterior cingulate, caudal anterior cingulate, superior frontal, and temporal pole) signatures are adjusted for the region of interest's surface areas.

^d^ Bonferroni corrected *p*‐value (*α*/*n* [0.05/4]) = ≥ 0.013.

**TABLE 4:** Associations of brain age gap with neuroimaging markers of vascular brain injury.

	Model 1^a^		Model 2^b^	
Parameter	β (95% CI)	*p*‐value	β (95% CI)	*p*‐value
SVD score				
0	Reference		Reference	
1	0.07 (−0.19, 0.32)	0.617	−0.13 (−0.39, 0.12)	0.302
2	0.43 (0.04, 0.81)	0.029	0.12 (−0.27, 0.51)	0.540
≥3	1.08 (0.51, 1.65)	<0.001	0.14 (−0.49, 0.76)	0.664
Fractional anisotropy	−20.2 (−24.9, −15.5)	<0.001	−21.1 (−26.2, −16.1)	<0.001

Abbreviations: CI, confidence interval; SVD, small vessel disease.

^a^ Model 1: Robust linear regression models included the SVD score and fractional anisotropy separately.

^b^ Model 2: Robust linear regression model included SVD score and fractional anisotropy simultaneously and biological sex.

Among the SVD markers, sex‐adjusted RLRMs revealed separate associations of WMHV (β = 0.05 [95% CI 0.03, 0.06], *p* < 0.001), greater WMHs burden (β for confluent = 0.49 [95% CI 0.19, 0.79], *p* = 0.001), lacunes (β = 0.45 [95% CI 0.03, 0.86], *p* = 0.037), and large infarcts (β = 2.23 [95% CI 1.25, 3.21], *p* < 0.001) with greater BAG (Table S3). After simultaneous adjustment for all cerebrovascular markers, only WMHV remained associated with BAG (β = 0.08 [95% CI 0.06, 1.0], *p* < 0.001).

### 3.4 Associations of BAG with inflammatory, glucose, and lipids biomarkers

RLRMs for inflammation, glucose, and lipid biomarkers indicate that BAG was associated with high CRP (β = 0.54 [95% CI 0.13 0.96], *p* = 0.011) and elevated glucose (β = 0.46 [95% CI 0.11, 0.81], *p* = 0.011) levels (Table 5). These associations remained similar after adjusting for sex.

**TABLE 5:** Associations of brain age gap with inflammatory, glucose, and lipids biomarkers.

Plasma biomarkers^c^	Model 1^a^		Model 2^b^	
	β (95% CI)	*p*‐value	β (95% CI)	*p*‐value
C‐reactive protein				
Normal (< 5 mg/L)	Reference		Reference	
Altered (≥ 5 mg/L)	0.26 (−0.06, 0.58)	0.114	0.25 (−0.07, 0.57)	0.124
Low (≥ 5–7 mg/L)	−0.09 (−0.56, 0.37)	0.694	−0.16 (−0.64, 0.33)	0.527
High (≥ 8 mg/L)	0.51 (0.10, 0.93)	0.015	0.54 (0.13, 0.96)	0.011
Homocysteine				
Normal (< 16 µmol/L)	Reference		Reference	
High (≥16 µmol/L)	0.10 (−0.18, 0.39)	0.476	0.11 (−0.19, 0.41)	0.464
Glucose				
Normal (< 7.0/11.1 mmol/L fasting/non‐fasting)	Reference		Reference	
High (≥7.0/11.1 mmol/L fasting/non‐fasting)	0.43 (0.10, 0.76)	0.010	0.46 (0.11, 0.81)	0.011
Total cholesterol				
Normal (< 7.8 mmol/L)	Reference		Reference	
High (≥7.8 mmol/L)	0.15 (−0.40, 0.70)	0.594	0.26 (−0.51, 1.03)	0.504
Triglycerides				
Normal (< 2.6 mmol/L)	Reference		Reference	
High (≥**2.6** mmol/L)	0.45 (−0.09, 0.98)	0.100	0.28 (−0.32, 0.87)	0.363
High‐density cholesterol				
Normal (> 1.0 mmol/L)	Reference		Reference	
Reduced (≤1.0 mmol/L)	0.18 (−0.22, 0.58)	0.368	0.09 (−0.36, 0.53)	0.697
Low‐density cholesterol				
Normal (<5.3 mmol/L)	Reference		Reference	
High (≥5.3 mmol/L)	−0.07 (−0.55, 0.42)	0.789	−0.24 (−0.92, 0.44)	0.483

Abbreviation: CI, confidence interval.

^a^ Model 1: Robust linear regression models included each biomarker separately.

^b^ Model 2: Robust linear regression model included all biomarkers simultaneously and biological sex.

^c^ Cutoffs were used according to the Sahlgrenska University Hospital laboratory values, except for glucose where the American Diabetes Association recommended cutoffs applied. In participants with abnormal C‐reactive protein, the continuous C‐reactive protein levels were dichotomized according to the median value into low (5–7 mg/L) and high (≥ 8 mg/L).

### 3.5 Associations of BAG with cognitive function

Education‐adjusted RLRMs showed that greater BAG was associated with poor cognitive performance (β for global cognition = −0.05 [95% CI −0.07, −0.03], *p* < 0.001). This was also observed for attention/speed and visuospatial abilities (Table 6, model 1; Bonferroni‐adjusted *p* ≥ 0.008). After sex adjustment, the association between BAG and executive function became statistically significant (β = −0.05 [95% CI −0.08, −0.02], *p* = 0.001), suggesting a possible modifying role of sex (Table 6, model 2).

**TABLE 6:** Associations between cognitive function and brain age gap.

	Model 1^a^		Model 2^b^	
Cognitive function (outcome)	β (95% CI)	*p*‐value^c^	β (95% CI)	*p*‐value^c^
Global cognition	−0.05 (−0.07, −0.03)	<0.001	−0.05 (−0.07, −0.03)	<0.001
Episodic memory	−0.04 (−0.08, 0.01)	0.121	−0.03 (−0.06, −0.01)	0.013
Attention/speed	−0.06 (−0.10, −0.02)	0.008	−0.05 (−0.08, −0.02)	0.002
Executive function	−0.05 (−0.10, −0.01)	0.047	−0.05 (−0.08, −0.02)	0.001
Verbal fluency	−0.06 (−0.11, −0.02)	0.009	−0.04 (−0.07, −0.004)	0.028
Visuospatial abilities	−0.09 (−0.14, −0.03)	0.003	−0.06 (−0.10, −0.02)	0.001

Abbreviation: CI, confidence interval.

^a^ Model 1: Robust linear regression models included each cognitive outcome separately, BAG as independent variable, and were adjusted for education.

^b^ Model 2: Model 1 + biological sex.

^c^ Bonferroni corrected *p*‐value (*α*/*n* [0.05/6]) = ≥ 0.008.

### 3.6 Stratified analysis by biological sex

**FIGURE 2:**
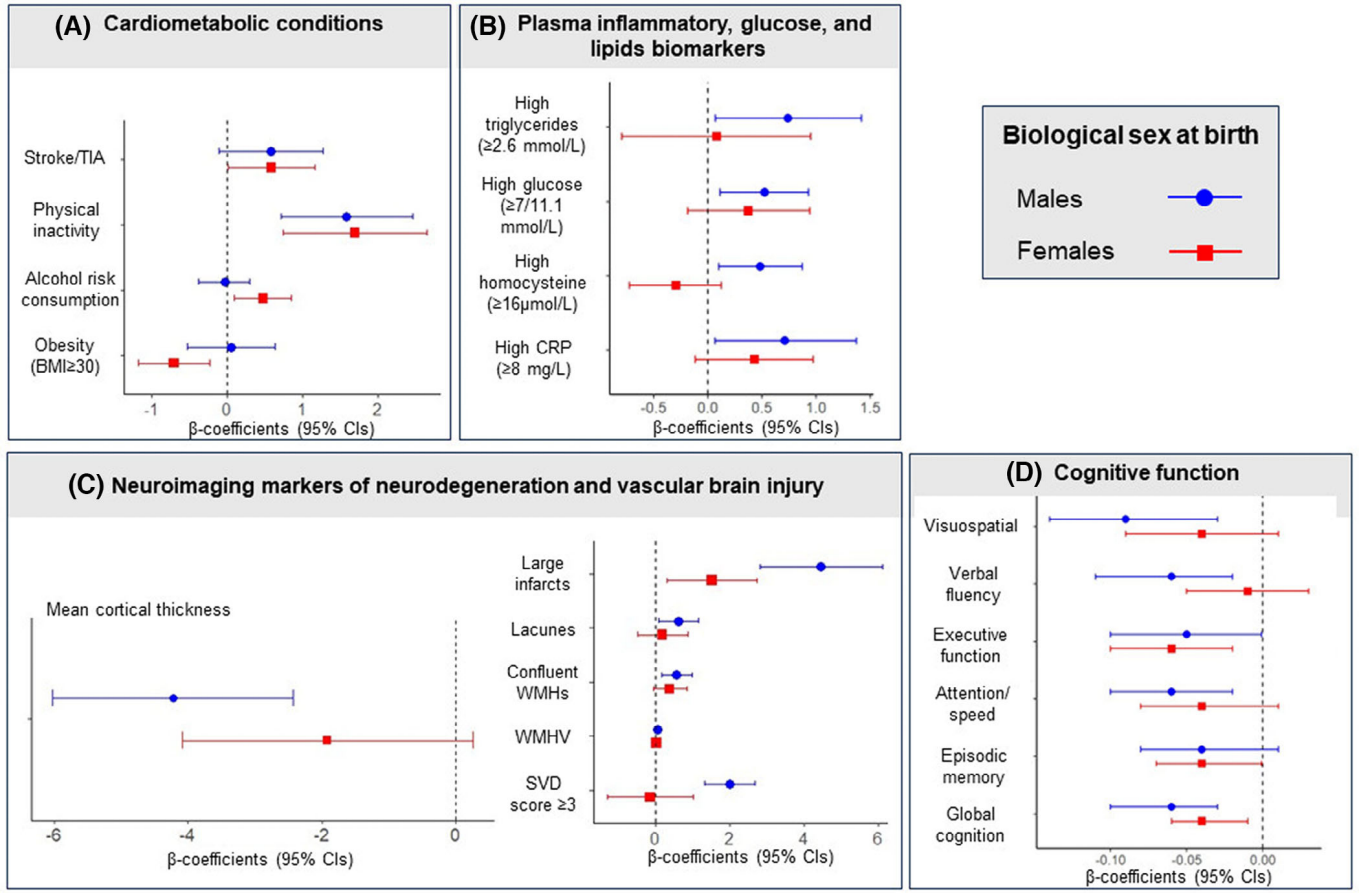
Sex‐specific associations of brain age gap with life exposures, biological processes, brain injury, and cognitive outcomes. The figure displays robust linear regression models’ β‐coefficients and 95% CIs for the associations of brain age gap with cardiometabolic conditions (panel A); plasma inflammatory, glucose, and lipids biomarkers (panel B), neuroimaging markers of neurodegenerative and vascular brain injury (panel C), and cognitive outcomes (panel D) in males (blue circle) and females (red square). All models displayed in Tables 2, 3, 4, 5, 6 were repeated in stratified analysis by biological sex at birth (i.e., among males and females) to identify sex‐specific factors related to the brain age gap. In panels A–C: Brain age gap was used as dependent variable (outcome). In panel D, cognitive composite scores were used as dependent variables in separate education‐adjusted robust linear regression models, whereas the brain age gap was entered as independent variable. BMI, body mass index; CI, confidence interval; CRP, C‐reactive protein.

To examine potential sex differences in BAG‐associated factors, we repeated RLRMs among males and females. Figure 2 provides a concise summary of the main findings.

Among females, greater BAG was associated with alcohol risk consumption (β = −0.47 [95% CI 0.09, 0.84], *p* = 0.016) and stroke/TIA (β = −0.58 [95% CI 0.01, 1.15], *p* = 0.047), whereas obesity was associated with lower BAG. Among males, increasing BAG was linked to lower mean cortical thickness (β = −4.22 [95% CI −6.02, −2.43], *p* < 0.001), SVD score ≥3 (β = 2.01 [95% CI 1.34, 2.67], *p* < 0.001)—especially, confluent WMHs and lacunes—and altered inflammation, glucose, and lipid biomarkers.

Cognitively, greater BAG was associated with lower episodic memory (β = −0.04 [95% CI −0.07, −0.001], *p* = 0.040) in females but not among males. Similarly, males exhibited specific associations between BAG and attention/speed (β = −0.06 [95% CI −0.10, −0.02], *p* = 0.008), verbal fluency (β = −0.06 [95% CI −0.11, −0.02], *p* = 0.009), and visuospatial abilities (β = −0.09 [95% CI −0.14, −0.03], *p* = 0.003) that were not observed in females (Figure 2, panel C).

In both sexes, BAG remained associated with physical inactivity, lower white matter structural integrity, greater WMHV, large infarcts, poorer global cognition, and executive function.

### 3.7 Supplementary analysis

No statistically significant associations were detected between BAG and biomarkers of AD pathology, neurodegeneration, and BBB in the CSF subsample (Table S4).

We assessed whether participants with prediabetes had as favorable lifestyle factors as those with normoglycemia. Individuals with prediabetes showed higher alcohol risk consumption (25.8% vs. 36.8%, Pearson *χ* ^2^ = 0.003). No differences were observed in terms of physical inactivity or smoking. Additionally, we attempted to control for potential reverse causality by including only participants with MMSE score > 27 (*n* = 724). This exclusion did not affect the observed associations, except for prediabetes that become borderline associated with BAG (β = −0.23 [95% CI −0.48, 0.01], *p* = 0.063), although the magnitude and direction of β‐coefficient were like the initial analysis, indicating a loss of power.

## 4 DISCUSSION

In this population‐based cross‐sectional study of septuagenarians, findings highlight that physical inactivity, diabetes, and stroke/TIA were independently associated with higher BAG, reflecting older‐appearing brains. Conversely, prediabetes was associated with younger‐appearing brains (lower BAG). Regular physical activity moderated the obesity‐BAG relationship, yielding the lowest BAG in individuals with obesity who were physically active. Greater cortical thickness, particularly in AD‐ and resilience‐related regions, was linked to lower BAG. Conversely, a higher burden of SVD, white‐matter microstructural alterations, systemic inflammation, and high blood glucose levels were associated with a greater BAG, highlighting their influence on brain health in late life. Greater BAG was also related to poorer cognitive outcomes, particularly attention/speed and visuospatial abilities. Notably, sex‐specific associations emerged, suggesting distinct pathological and resilience pathways to cognitive disorders between females and males. Together, these findings confirm that vascular‐related lifestyles and health factors likely contribute to shaping the appearance of the brain during the aging process. The interplay between vascular brain injury, inflammation, and insulin‐related dysregulations may be the key to understanding the neurobiological underpinnings of BAG, therefore, of resilience mechanisms in aging.

Our study adds to the growing evidence highlighting the crucial role of CMDs in late‐life brain health. Stroke, diabetes, and physical inactivity are well‐established risk factors for various brain and cognitive outcomes.^1^, ^33^ Artificial intelligence (AI)‐driven algorithms predicting the physiological brain age offers valuable insights into brain health, allowing to identify modifiable factors associated with atypical aging processes, that is, deviations from chronological age (BAG).^15^, ^25^, ^34^ Our findings align with prior limited research linking increased BAG to stroke,^21^, ^35^ diabetes,^21^ or physical activity,^36^, ^37^ despite reporting mixed results.^21^, ^38^ Greater BAG was associated with a greater burden of SVD and alterations in white‐matter microstructure, supporting their key contribution to brain atrophy.^1^ These vascular‐related tissue injuries have been proposed as the primary structural link between CMDs and related cognitive disorders, that is, vascular cognitive impairment (VCI),^39^, ^40^ where genetic predisposition and cardiometabolic risk factors are hypothesized to drive a cascade of biological processes leading to cerebrovascular disease and cognitive deficits, particularly in processing speed, attention, and visuospatial abilities—the cognitive correlates of greater BAG in our study.^1^, ^40^ Demyelination is a key contributor to brain atrophy. CMDs and related features such as insulin‐resistance and central adiposity, have been associated with myelin alterations in older adults.^41^, ^42^ In contrast, physical activity and cardiorespiratory fitness have shown neuroprotective effects on myelin integrity.^43^ Therefore, myelin may be a critical link between CMDs, vascular brain injury, and cognitive disorders, warranting further investigation.

Our findings also revealed an association between prediabetes and lower BAG, suggesting that slightly elevated glucose levels could favor brain preservation. However, this became statistically not significant after adjustment for all risk factors simultaneously. The relationship between prediabetes and brain aging remains elusive, with some studies linking prediabetes to SVD, particularly WMHs.^3^, ^44^, ^45^ Lifestyle modifications, such as smoking cessation, adoption of healthy diets (e.g., low‐fat and high fiber), regular physical activity, and weight loss, typically used to manage prediabetes, may improve glycemic control and positively impact brain health.^46^ In our cohort, individuals with prediabetes showed similar lifestyles to those with normoglycemia (e.g., physical activity, smoking), except for higher alcohol consumption. Future longitudinal studies with detailed lifestyle information (e.g., physical exercise, diet, weight changes) should examine the role of behavioral changes and glucose variability in brain aging and BAG. The beneficial impact of favorable lifestyle in mitigating the effect of established dementia risk factors is further highlighted by the fact that participants with obesity (sharing insulin‐resistance features with prediabetes) engaging in regular physical activity had the lowest BAG (younger brains), aligning with evidence that lifestyle interventions can offset the negative effects of vascular risk factors on brain health.^47^, ^48^

The relative preservation of the brain integrity throughout life is referred to as brain maintenance.^49^ Brain maintenance and cognitive reserve are the primary resilience mechanisms that help explain why cognition is preserved into older ages in some individuals but not others.^12^ In our study, greater cortical thickness, particularly in AD and resilience‐related regions, was associated with lower BAG, implying younger‐appearing brains exhibit less atrophy along the BAG continuum. However, questions persist about whether the opposite end of the continuum, characterized by older brains with more pronounced atrophy, reflects accelerated brain aging or if some individuals experience minimal cognitive decline, suggesting exceptional coping mechanisms linked to cognitive reserve.

Inflammation and insulin‐related dysregulations could be key biological pathways linking life exposures to SVD and VCI.^1^, ^7^ CMDs often increase inflammation and metabolic dysfunction, both of which are associated with SVD.^5^, ^50^, ^51^ We have previously shown that heightened inflammation coupled with elevated glycated hemoglobin escalated dementia risk in older adults.^10^ Our current results further link low‐grade inflammation (estimated with CRP) and elevated blood glucose with a greater BAG. While inflammation is a natural part of aging and serves a beneficial role in bodily tissue maintenance and repair, low‐grade inflammation can become detrimental to health.^52^ Emerging studies have linked various inflammatory markers (e.g. CRP, interleukins, tumor necrosis factor alpha) to SVD,^7^ AD,^6^ and cognitive disorders.^53^, ^54^ More recently, Hayek and coll.^55^ showed that inflammatory signatures characterized by proinflammatory molecules (e.g., CRP, interleukin‐8) were associated with lower subcortical gray matter volume as well as lower frontal white matter volumes, whereas inflammatory signatures related to neuronal and vessels protection was associated with structural brain preservation (i.e., brain maintenance). This evidence suggests that not only inflammation, but likely also metabolic processes, work synergistically in influencing BAG, and future studies should examine this interaction further.

Notably, we found no association between CSF biomarkers for AD/neurodegeneration and BAG. Limited research exists on this relationship in cognitively unimpaired adults, with one study reporting associations between higher BAG and abnormal amyloid‐β levels,^56^ while another found none.^57^ However, Doering et al.^57^ identified a correlation between higher BAG and lower amyloid‐β levels in individuals with subjective cognitive decline and mild cognitive impairment, suggesting that BAG may serve as marker of AD pathology/risk once cognitive symptoms emerge.

Sex differences in factors associated with BAG were evident in our study. Among males, greater BAG was linked to cortical thinning, greater SVD burden, inflammation, alterations in glucose and triglycerides plasma levels, and poor cognitive performance in attention/speed, verbal fluency, and visuospatial abilities. Such associations were not evident in females, where greater BAG was associated with worse executive function. Emerging literature suggests that pathological pathways leading to dementia may differ between sexes, with women exhibiting greater vulnerability to AD^58^ and cerebrovascular pathology after menopause.^59^ It has been suggested that female reproductive factors, such as estrogen exposure across the reproductive lifespan and after menopause, may provide protective effects for vascular and cognitive health in later life.^60^, ^61^ Future research should focus on understanding the underlying factors and mechanisms that contribute to BAG differences between sexes.

Our study's primary strength lies in the comprehensive examinations including the investigation of risk/protective factors, biological processes, structural brain alterations, and cognitive performance associated with the BAG, a potential resilience biomarker, considering sex differences. Additional strengths encompass a well‐characterized, sizable, population‐based brain MRI sample, uniformly aged 70, reducing age‐related bias. Integration of clinical and biomarkers data from various sources (i.e., medical history alongside objective measurements) enabled a comprehensive identification of risk/protective factors, minimizing misclassification. However, the cross‐sectional design restricted our ability to discern temporal–causal relationships among life exposures, structural brain alterations, biological processes, and BAG. Longitudinal studies are warranted to elucidate these interactions along this chain of events. Also, cross‐sectional brain age could capture differences between individuals (not within) in brain aging, which may reflect early‐life influences on brain structure rather than the actual rate of brain change in late life.^62^ Reliance on self‐reported lifestyle measures (e.g., physical activity) may introduce recall bias, particularly for participants at an earlier stage of cognitive deterioration. Nonetheless, our sample included individuals without dementia, with a median MMSE score of 29, and consistent results were obtained even when analyses were restricted to those with MMSE scores > 27. Obesity was defined using BMI, a crude anthropometric measure that cannot differentiate between muscle and fat mass. Future studies with direct body composition measures (i.e., dual‐energy X‐ray absorptiometry [DEXA]) are needed to clarify the interaction between physical activity and obesity on brain aging. Finally, we were unable to address the contribution of genetic and functional risk factors to BAG.

## 5 CONCLUSIONS

Our study emphasizes the importance of CMDs (i.e., insufficient physical activity, obesity, diabetes, prediabetes, and stroke), inflammation, insulin‐related processes, cerebrovascular disease (i.e., greater SVD burden, particularly WMHs, lacunes, large infarcts, and white‐matter microstructural abnormalities) for deviations from “expected” CA, that is, BAG. However, such influence may not be irreversible as it could be mitigated by lifestyle changes aimed at preserving brain structure. Lifestyle interventions are needed to determine whether targeting physical activity can mitigate or even reverse the adverse effects of obesity on the brain. Consistent with a vascular‐driven hypothesis, BAG was associated with cognitive dysfunction typically seen in VCI, underscoring the importance of managing vascular health in late life. Finally, sex differences in BAG‐related factors advocate for further research to elucidate the driving factors.

## CONFLICT OF INTEREST STATEMENT

S.K. has served on scientific advisory boards, speaker and/or as consultant for Roche, Geras Solutions, Optoceutics, Eli Lilly, Biogen and Bioarctic. The remaining authors have nothing to disclose. Author disclosures are available in the Supporting Information.

## CONSENT STATEMENT

All participants provided written informed consent, and in cases where an individual was unable to provide consent, this was obtained from their next‐of‐kin.

## Supporting information



Supporting Information

Supporting Information
