# Supplementary material for: Biological brain age and resilience in cognitively unimpaired 70‐year‐old individuals
Source: Alzheimers Dement. 2024 Dec 20;21(2):e14435. doi: 10.1002/alz.14435 (PMC11848408; doi:10.1002/alz.14435)
Supplement: Supplementary file 1 — Supporting Information [file ALZ-21-e14435-s001.docx]

**SUPPLEMENTARY MATERIAL**

**Supplementary Figure 1.** Schematic representation of the study hypothesis and analytical approach.

**Supplementary Figure 2.** Flow chart of the study population.

**Supplementary Table 1.** Assessment of medical conditions and data sources in the H70-1944 Birth Cohort.

**Supplementary Table 2.** Joint effect analysis for obesity and physical activity in relation to the Brain Age Gap.

**Supplementary Table 3.** Associations of Brain Age Gap with MRI markers of vascular brain injury.

**Supplementary Table 4.** Associations of Brain Age Gap with CSF biomarkers of neurodegeneration, Alzheimer’s disease, and BBB integrity in the subsample with lumbar puncture.

**Supplementary Figure 1.** Schematic representation of the study hypothesis and analytical approach.


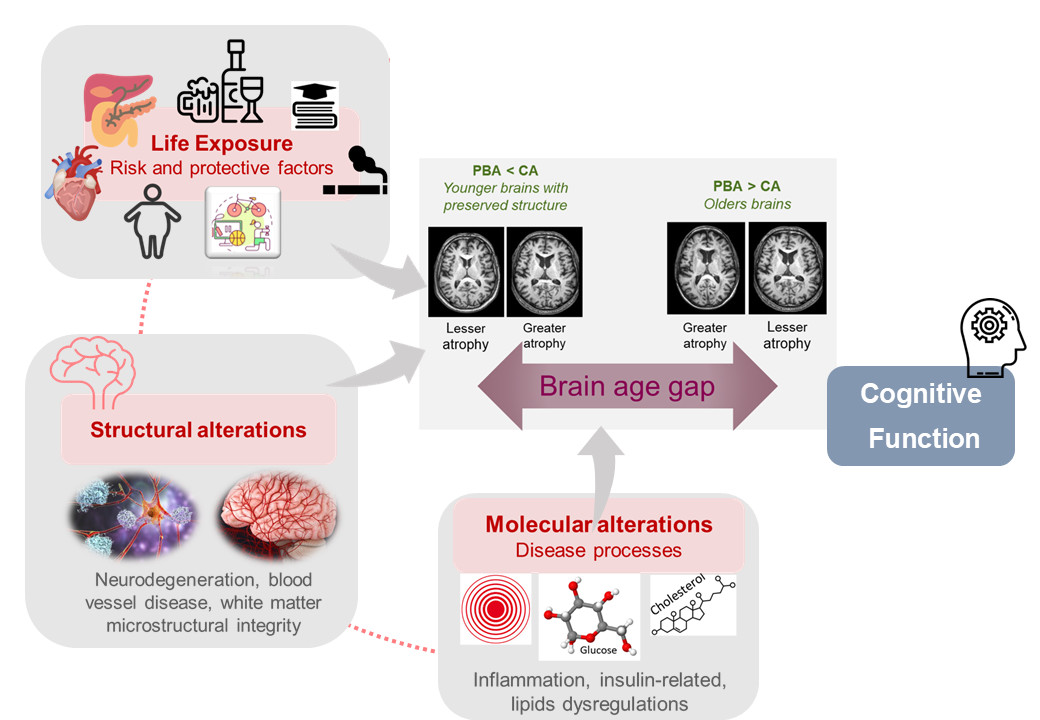


Our conceptual framework suggests that resilience is influenced by the interactions of risk and protective factors (life exposures, e.g., cardiometabolic risk factors and disorders, lifestyle habits), neurodegenerative and cerebrovascular disease affecting brain blood vessels (structural alterations), and shared biological processes—particularly inflammation, insulin-related, and lipid dysregulations (molecular alterations). These interactions play a critical role in determining the brain age gap (BAG), the difference between biological brain age and chronological age. BAG is conceptualized as existing on a continuum, from younger- to older-looking brains, with distinct impacts on cognitive function. To start testing this hypothesis, in this study we assessed if these elements are linked to BAG, and if so, in what way. Therefore, the direction of the grey arrows explains the rationale behind the choice of exposures and outcomes in our analysis; however, they do not represent temporality for the current study.

**Supplementary Figure 2.** Flow chart of the study population.

**
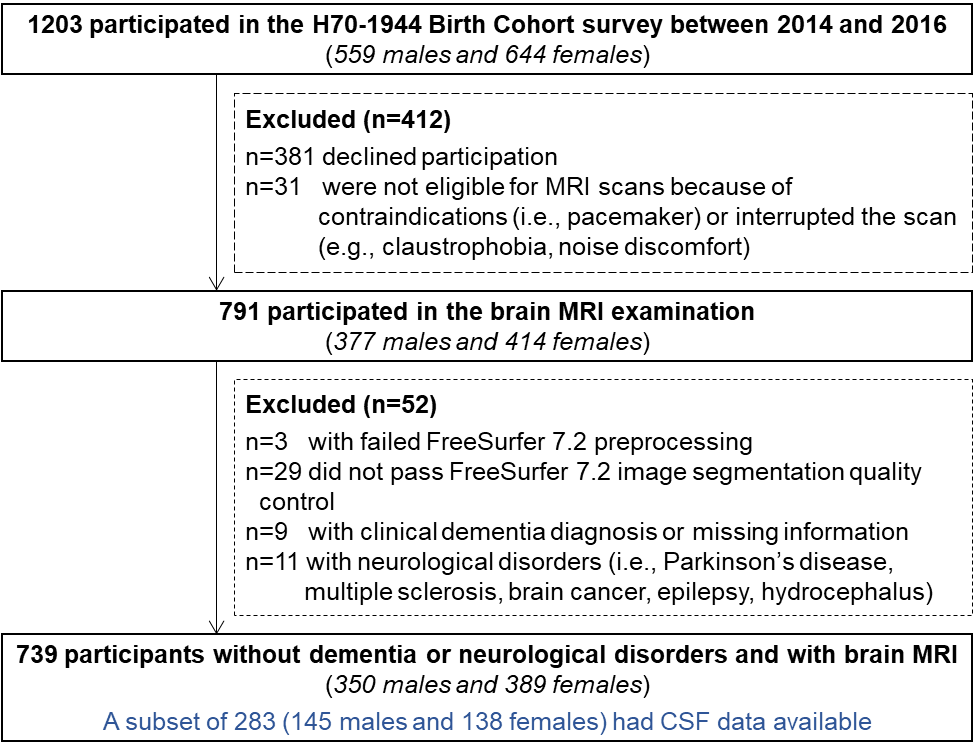
**

**Abbreviations:** CSF, cerebrospinal fluid; MRI, Magnetic resonance imaging.

**Supplementary Table 1.** Assessment of medical conditions and their data sources in the H70-1944 Birth Cohort.

|  | **Assessment and definitions** | **Data sources** |
| --- | --- | --- |
| **Hypertension**  (no vs. yes) | World Health Organization 2023 and International Society of Hypertension * | Self-reported history, use of antihypertensive medication, and/or altered blood pressure (SBP/DBP ≥140/90 mmHg; measured in the right arm in the sitting position after 5 minutes rest using a manual sphygmomanometer). |
| **Heart disease**  (no vs. yes) | Included the presence of myocardial infarction, angina pectoris, heart failure, and/or atrial fibrillation. | A standard 12-lead ECG was coded, according to the Minnesota Code, by a biomedical analyst working at the cardiac laboratory at Sahlgrenska University Hospital.  **Myocardial infarction (no, yes)**  Self-reported history, presence of moderate or major Q-waves on ECG (Minnesota Codes: 1-1-X or 1-2-X, excluding 1-2-6 and 1-2-8)  **Angina pectoris (no, yes)**  Self-reported history or standard criteria †  **Heart failure (no, yes)**  Self-reported history  **Atrial fibrillation (no, yes)**  Self-reported history or ECG (Minnesota Code: 8-3) |
| **Diabetes status**  (normoglycemia vs. prediabetes, and diabetes) | American Diabetes Association standards 2023 ‡ | **Normoglycemia**  Absence of diabetes and prediabetes, and glucose level <5.6 (if fasting for ≥8 hours) or <7.8 mmol/L.  **Prediabetes**  Absence of diabetes and blood glucose level ≥5.6-6.9 (if fasting for ≥8 hours) or ≥7.8-11.0 (no fasting or missing fasting status) mmol/L.  **Diabetes**  Self-reported history, use of antidiabetic medication, or blood glucose levels ≥7.0 (if fasting for ≥8 hours) or ≥11.1 (no fasting or missing fasting status) mmol/L. |
| **Stroke/TIA**  (no vs. yes) | Sudden onset of focal symptoms or aphasia lasting for more than 24 hours (stroke) or less than 24 hours (TIA) | Self-report, key-informants interview, or the national patient register (ICD-10 codes: I60. I61. I629. I630–I635. I638–I639. I64. I690–I691. and I693–I694). |
| **Depression**  (no vs. minor/major) | DSM-5th editions criteria. | Computerized symptom algorithms based on clinical assessments of depressive symptoms within the CPRS §. |

Abbreviations: CPRS, Comprehensive Psychopathological Rating Scale; ECG, electrocardiogram; DBP, diastolic blood pressure; DSM, Diagnostic and Statistical Manual of Mental Disorders; ICD, International Classification of Disease; SBP, systolic blood pressure; TIA, transient ischemic attack.

* Unger et al. 2020 International Society of Hypertension Global Hypertension Practice Guidelines. Hypertension 2020;75:1334-1357.

† Rose. The diagnosis of ischaemic heart pain and intermittent claudication in field surveys. Bull World Health Organ 1962;27:645-58;

‡ ElSayed et al on behalf of the American Diabetes Association. 2. Classification and Diagnosis of Diabetes: Standards of Care in Diabetes-2023. Diabetes Care 2023 ;46(Suppl 1):S19-S40.

§ Rydberg Sterner et al. Depression in relation to sex and gender expression among Swedish septuagenarians. PLoS One 2020;15:e0238701.

**Supplementary Table 2.** Joint effect analysis for obesity and physical activity in relation to the Brain Age Gap.

| **Joint effect** | | **n** | | **Multi-adjusted robust linear regression model *** | | | |
| --- | --- | --- | --- | --- | --- | --- | --- |
|  |  |  |  | **β (95% CI)** | | **p** | |
| **Physical activity** | **Obesity** | |  | |  | |  |
| Inactive | No | | 6 | | Reference | |  |
| Active | No | | 295 | | -1.45 (-2.67, -0.23) | | 0.020 |
| Inactive | Yes | | 11 | | 0.01 (-1.47, 1.48) | | 0.999 |
| Active | Yes | | 25 | | -1.92 (-3.15, -0.69) | | 0.002 |

* Robust linear regression model was adjusted for biological sex, education, smoking, alcohol risk consumption, hypertension, heart disease, diabetes status, stroke/TIA, depression, and apolipoprotein-ɛ4 allele.

**Supplementary Table 3.** Associations of Brain Age Gap with MRI markers of vascular brain injury.

| **MRI markers** | **Model 1** * | | **Model 2** † | |
| --- | --- | --- | --- | --- |
|  | **β (95% CI)** | ***p*** | **β (95% CI)** | ***p*** |
| WMH volume (mm^3^) | 0.05 (0.03, 0.06) | <0.001 | 0.05 (0.03, 0.06) | <0.001 |
| WMHs burden |  |  |  |  |
| None/punctate | Reference |  | Reference |  |
| Confluent | 0.48 (0.18, 0.78) | 0.002 | 0.49 (0.19, 0.79) | 0.001 |
| Lacunes |  |  |  |  |
| No | Reference |  | Reference |  |
| Yes | 0.42 (0.01, 0.84) | 0.047 | 0.45 (0.03, 0.86) | 0.037 |
| PVS centrum semiovale |  |  |  |  |
| No (0-11) | Reference |  | Reference |  |
| Yes (≥11-40) | 0.07 (-0.17, 0.32) | 0.555 | 0.08 (-0.17, 0.32) | 0.545 |
| PVS basal ganglia |  |  |  |  |
| No (0-11) | Reference |  | Reference |  |
| Yes (≥11-40) | 0.23 (-0.03, 0.49) | 0.083 | 0.24 (-0.02, 0.50) | 0.073 |
| Cerebral microbleeds |  |  |  |  |
| No | Reference |  | Reference |  |
| Yes | -0.01 (-0.36, 0.34) | 0.949 | -0.01 (-0.36, 0.34) | 0.958 |
| Large infarcts (>15 mm) |  |  |  |  |
| No | Reference |  | Reference |  |
| Yes | 2.25 (1.28, 3.23) | <0.001 | 2.23 (1.25, 3.21) | <0.001 |

Abbreviations: CI, confidence interval; WMH, white matter hyperintensity; PVS, perivascular spaces.

* Model 1: Robust linear regression models included each MRI marker separately.

† Model 2: Robust linear regression model included all MRI markers simultaneously + biological sex.

**Supplementary Table 4**. Associations of Brain Age Gap with CSF biomarkers of neurodegeneration, Alzheimer’s disease, and BBB integrity in the subsample with lumbar puncture.

| **CSF biomarkers (for n=283 participants)** ‡ | **Model 1** * | | **Model 2** † | |
| --- | --- | --- | --- | --- |
|  | **β (95% CI)** | ***p*** | **β (95% CI)** | ***p*** |
| β-amyloid 42 |  |  |  |  |
| Normal (> 530 pg/mL) | Reference |  | Reference |  |
| Altered (≤ 530 pg/mL) | -0.28 (-0.61, 0.05) | 0.091 | -0.29 (-0.62, 0.04) | 0.083 |
| P-tau (ng/L) |  |  |  |  |
| Normal (< 80 pg/mL) | Reference |  | Reference |  |
| Altered (≥ 80 pg/mL) | 0.46 (-0.25, 1.18) | 0.200 | 0.46 (-0.25, 1.18) | 0.200 |
| Total tau (ng/L) |  |  |  |  |
| Normal (<350 pg/mL) | Reference |  | Reference |  |
| Altered (≥ 350 pg/mL) | 0.13 (-0.23, 0.48) | 0.487 | 0.11 (-0.25, 0.47) | 0.548 |
| Neurofilament light (pg/mL)§ |  |  |  |  |
| Low [T1: ≥255-616] | Reference |  |  |  |
| Middle [T1: ≥617-849] | -0.19 (-0.60, 0.21) | 0.348 | -0.20 (-0.61, 0.20) | 0.321 |
| High [T1: ≥850-6233] | -0.05 (-0.45, 0.36) | 0.814 | -0.07 (-0.48, 0.34) | 0.738 |
| Neurogranin (pg/mL) **^d^** |  |  |  |  |
| Low [T1: ≥54-173] | Reference |  | Reference |  |
| Middle [T1: ≥173-220] | -0.02 (-0.42, 0.39) | 0.938 | -0.01 (-0.41, 0.40) | 0.967 |
| High [T1: ≥220-451] | 0.14 (-0.27, 0.54) | 0.506 | 0.14 (-0.26, 0.55) | 0.488 |
| CSF/serum albumin ratio |  |  |  |  |
| Normal (< 10.2) | Reference |  | Reference |  |
| Altered (≥10.2) | -0.05 (-0.63, 0.52) | 0.855 | -0.10 (-0.69, 0.48) | 0.729 |

Abbreviations: AD, Alzheimer’s disease; CI, confidence interval; CSF, cerebrospinal fluid.

* Model 1: Robust linear regression models included each biomarker separately.

† Model 2: Robust linear regression model included all biomarkers simultaneously + biological sex.

‡ Cut-offs were used according to the Sahlgrenska University Hospital laboratory reference values.

§ Neurogranin and neurofilament light were divided into tertiles, with the highest tertile (T3) reflecting higher biomarker levels.
